# Special Issue “Atherosclerosis 2: From Molecular Mechanisms and Pathophysiology to Novel Therapeutic Approaches”

**DOI:** 10.3390/ijms26178515

**Published:** 2025-09-02

**Authors:** Antonio Barbato

**Affiliations:** Department of Clinical Medicine and Surgery, University of Naples Federico II, 80131 Naples, Italy; abarbato@unina.it

Atherosclerosis is a chronic inflammatory disease that affects medium- and large-sized arteries and remains a leading cause of global morbidity and mortality [1]. It is characterized by the accumulation of lipids, inflammatory cells, and fibrous tissue in the arterial wall, leading to the formation of atherosclerotic plaques [2]. In addition to well-known factors (e.g., high blood pressure, dyslipidemia, diabetes, and excess body weight), there are several emerging factors associated with atherosclerosis, such as inflammation, endothelial dysfunction, intestinal microbiota alteration, uric acid, vitamin D, or miRNA expression, which could potentially explain the residual cardiovascular risk [3].

This Special Issue integrates key findings from recent original investigations and review articles to present a multifaceted view of the current landscape in atherosclerosis research. A brief introduction to the main innovations and limitations emerging from this Special Issue is provided below.

The foundation of vascular health lies in the endothelium, and its phenotype can vary based on anatomical location. Frolov, A. et al. [4] employed a multi-omics approach to compare endothelial cells from the human coronary artery (HCAEC) and the internal thoracic artery (HITAEC), the latter being a common graft in coronary bypass surgery. They identified significant molecular heterogeneity: HCAECs exhibited a profile oriented toward extracellular matrix construction, while HITAECs showed greater pro-inflammatory activity. Intriguingly, despite these molecular differences, no specific protein markers were found that functionally distinguished the two cell types in vitro. A notable limitation included the inability to compare the proteomic data under laminar flow and static conditions, as the amount of total protein extracted from endothelial cells cultured in flow chambers was insufficient.

Endothelial dysfunction is a critical early event in atherosclerosis, often driven by factors like angiotensin II (Ang II). The molecular mediators of this process are under intense investigation. Dutka, M. et al. [5] demonstrated using a knockout mouse model that osteoprotegerin (OPG) is an essential factor for the development of Ang II-induced endothelial dysfunction. The novel observation that the absence of OPG exerts a protective effect suggests that OPG functions not merely as a biomarker but as an active mediator of the pathological process, as its deficiency is associated with reduced levels of pro-inflammatory cytokines within the vascular wall following Ang II stimulation. However, the translation of these findings to humans requires caution due to the complex regulation of OPG in human physiology.

Beyond biochemical stimuli, hemodynamic forces are central in determining sites of atherosclerotic plaque development. Cho, M. et al. [6] utilized a partial ligation mouse model of atherosclerosis and computational fluid dynamics (CFD) to analyze wall shear stress (WSS). Their innovative contribution was analyzing the standard deviation of time-averaged WSS as a new metric to correlate the heterogeneity of shear stress with plaque progression and inflammation. This approach provides a more nuanced understanding of hemodynamics than mean WSS alone, though the model primarily focuses on shear stress, neglecting other contributing biological factors.

As atherosclerosis progresses, circulating monocytes infiltrate the subendothelial space and transform into lipid-laden foam cells, a hallmark of the disease. Henni Mansour, A.S. et al. [7] provided a detailed characterization of different experimental models of these “foamy” macrophages. Their novel analysis, using flow cytometry and two-photon Excited Fluorescence (TPEF) imaging, revealed that foam cells generated with oxidized LDL (Mox) exhibit a more inflammatory and dysfunctional profile than those generated with acetylated LDL (Mac). Furthermore, they showed that this pro-inflammatory state could be partially reversed with antioxidant and cytokine treatments, offering a potential pathway for therapeutic intervention. The inherent limitation, however, is that these in vitro models cannot fully capture the complexity of the plaque microenvironment in vivo.

The search for novel molecular players continues with Kathuria, I. et al. [8] identifying nidogen-2 (NID2), a basement membrane protein, as a harmful factor that promotes both hepatosteatosis and atherosclerosis in a mouse model. To study the effect of NID2 overexpression, Apoe−/− mice were used in this study. One group of Apoe−/− mice, designated as controls, received an intraperitoneal injection of saline, while another group was administered a single dose of a recombinant AAV expressing the human *NID2* gene under the ubiquitous cytomegalovirus promoter, to induce NID2 overexpression. The novelty of this study is to link NID2 overexpression to the inhibition of AMP-activated protein kinase (AMPK) activation in the liver, a key regulator of energy metabolism. A significant limitation was that NID2 was overexpressed systemically in this mice model, making it difficult to isolate the specific contribution of liver versus vascular cells to the observed atherosclerotic phenotype.

Translating molecular findings into clinical practice is the goal of the work of Iusupova, A.O. et al. [9], who conducted a clinical study analyzing microRNAs and inflammatory proteins in patients with different phenotypes of coronary artery disease. Their key finding was that miRNA-145 serves as an independent predictor of ischemia with non-obstructive coronary arteries (INOCA), highlighting its potential as a valuable diagnostic biomarker to distinguish this challenging patient group. The study’s conclusions are tempered by a relatively small sample size and a focus on a limited panel of biomarkers.

Several recent reviews have synthesized broader areas of research. Sohn, M. and Lim, S. [10] performed a systematic review with meta-analysis on the antiplatelet agent cilostazol, confirming its efficacy in reducing major adverse cardiovascular events (MACE), particularly stroke, with a lower bleeding risk than aspirin. They caution, however, that the evidence base is predominantly from East Asian populations, limiting generalizability.

Theofilis, P. et al. [11] provided a comprehensive overview of coronary plaque erosion, an important alternative mechanism of acute coronary syndrome distinct from rupture. Their review integrates the latest on molecular mechanisms (e.g., neutrophil involvement), advanced intracoronary imaging diagnostics such as optical coherence tomography or intravascular ultrasound–near-infrared spectroscopy, and emerging conservative treatment strategies.

Exploring the link between systemic inflammation and accelerated atherosclerosis, Kasher, M. et al. [12] reviewed evidence for glycoprotein acetyls (GlycA) as a novel biomarker. This study included original genetic analyses, suggesting GlycA is a causal mediator linking the inflammation of rheumatoid arthritis to atherosclerosis, partly through shared lipid pathways. However, the origin of genome-wide association studies (GWAS) data from European cohorts may affect the generalizability of these genetic findings.

Finally, Getz, G.S. & Reardon, C.A. [13] reviewed the site specificity of atherosclerotic lesions, drawing largely on murine studies. They present an updated synthesis moving beyond hemodynamics alone to include the roles of genetics, adaptive immunity, and perivascular adipose tissue.

## Conclusions and Future Directions

The current research landscape reflects a multi-pronged and increasingly sophisticated approach to understanding atherosclerosis. The integration of multi-omics, advanced imaging modalities, and large-scale genetic data is driving a critical shift from correlational observations toward establishing causal pathways. Despite these significant advances, several knowledge gaps persist, representing major difficulties in the effective management of the disease [14].

A recurring theme across contemporary research is the challenge of translating findings from simplified in vitro systems and animal models to the complex reality of human pathophysiology [15]. While essential for mechanistic discovery, these models often fail to capture the full spectrum of the disease’s heterogeneity. Key areas requiring deeper investigation include the precise molecular mechanisms governing plaque stability, the intricate cellular and metabolic interplay within the plaque microenvironment, and the influence of epigenetic regulators like microRNAs [16]. Furthermore, the impact of demographic factors such as sex and age on disease progression remains incompletely understood, limiting the development of truly personalized diagnostic and therapeutic strategies [17]. Clinically, there is still a pressing need for robust biomarkers for the early detection of vulnerable plaques and for more accurate, non-invasive risk stratification tools. By focusing on these strategic priorities, the scientific community can bridge the existing knowledge gaps and translate research breakthroughs into meaningful clinical innovations that reduce the global burden of atherosclerotic disease.

## References

[1] Björkegren J., Lusis A. (2022). Atherosclerosis: Recent developments. Cell.

[2] Kong P., Cui Z.Y., Huang X.F., Zhang D.D., Guo R.J., Han M. (2022). Inflammation and Atherosclerosis: Signaling Pathways and Therapeutic Intervention. Signal Transduct. Target. Ther..

[3] Bekbossynova M., Saliev T., Ivanova-Razumova T., Andossova S., Kali A., Myrzakhmetova G. (2025). Beyond Cholesterol: Emerging Risk Factors in Atherosclerosis. J. Clin. Med..

[4] Frolov A., Lobov A., Kabilov M., Zainullina B., Tupikin A., Shishkova D., Markova V., Sinitskaya A., Grigoriev E., Markova Y. (2023). Multi-Omics Profiling of Human Endothelial Cells from the Coronary Artery and Internal Thoracic Artery Reveals Molecular but Not Functional Heterogeneity. Int. J. Mol. Sci..

[5] Dutka M., Garczorz W., Kosowska A., Buczek E., Godek P., Wojakowski W., Francuz T. (2024). Osteoprotegerin Is Essential for the Development of Endothelial Dysfunction Induced by Angiotensin II in Mice. Int. J. Mol. Sci..

[6] Cho M., Hwang J.S., Kim K.R., Kim J.K. (2024). Wall Shear Stress (WSS) Analysis in Atherosclerosis in Partial Ligated Apolipoprotein E Knockout Mouse Model through Computational Fluid Dynamics (CFD). Int. J. Mol. Sci..

[7] Henni Mansour A.S., Ragues M., Brevier J., Borowczyk C., Grevelinger J., Laroche-Traineau J., Garaude J., Marais S., Jacobin-Valat M.-J., Gerbaud E. (2024). Phenotypic, Metabolic, and Functional Characterization of Experimental Models of Foamy Macrophages: Toward Therapeutic Research in Atherosclerosis. Int. J. Mol. Sci..

[8] Kathuria I., Prasad A., Sharma B.K., Aithabathula R.V., Ofosu-Boateng M., Gyamfi M.A., Jiang J., Park F., Singh U.P., Singla B. (2024). Nidogen 2 Overexpression Promotes Hepatosteatosis and Atherosclerosis. Int. J. Mol. Sci..

[9] Iusupova A.O., Pakhtusov N.N., Slepova O.A., Khabarova N.V., Privalova E.V., Bure I.V., Nemtsova M.V., Belenkov Y.N. (2024). MiRNA-34a, miRNA-145, and miRNA-222 Expression, Matrix Metalloproteinases, TNF-α and VEGF in Patients with Different Phenotypes of Coronary Artery Disease. Int. J. Mol. Sci..

[10] Sohn M., Lim S. (2024). The Role of Cilostazol, a Phosphodiesterase-3 Inhibitor, in the Development of Atherosclerosis and Vascular Biology: A Review with Meta-Analysis. Int. J. Mol. Sci..

[11] Theofilis P., Vlachakis P.K., Papanikolaou A., Karakasis P., Oikonomou E., Tsioufis K., Tousoulis D. (2024). Coronary Plaque Erosion: Epidemiology, Diagnosis, and Treatment. Int. J. Mol. Sci..

[12] Kasher M., Freidin M.B., Williams F.M.K., Cherny S.S., Ashkenazi S., Livshits G. (2024). Glycoprotein Acetyls Is a Novel Biomarker Predicting Cardiovascular Complications in Rheumatoid Arthritis. Int. J. Mol. Sci..

[13] Getz G.S., Reardon C.A. (2024). Insights from Murine Studies on the Site Specificity of Atherosclerosis. Int. J. Mol. Sci..

[14] Libby P. (2021). The changing landscape of atherosclerosis. Nature.

[15] Zhang Y., Fatima M., Hou S., Bai L., Zhao S., Liu E. (2021). Research methods for animal models of atherosclerosis (Review). Mol. Med. Rep..

[16] Xu S., Kamato D., Little P.J., Nakagawa S., Pelisek J., Jin Z.G. (2019). Targeting Epigenetics and Non-Coding RNAs in Atherosclerosis: From Mechanisms to Therapeutics. Pharmacol. Ther..

[17] Kerkhof P., Tona F. (2023). Sex Differences in Diagnostic Modalities of Atherosclerosis in the Macrocirculation. Atherosclerosis.

